# Consequences of prion strain mixtures: Indifference, competition, or collusion

**DOI:** 10.1371/journal.ppat.1013956

**Published:** 2026-02-11

**Authors:** Amanda L. Woerman, Jason C. Bartz

**Affiliations:** 1 Department of Microbiology, Immunology, and Pathology, Prion Research Center, Colorado State University, Fort Collins, Colorado, United States of America; 2 Department of Medical Microbiology and Immunology, School of Medicine, Creighton University, Omaha, Nebsraska, United States of America; National Institutes of Health, UNITED STATES OF AMERICA

## Introduction

Prion diseases are invariably fatal neurogenerative diseases affecting several species, including humans, with a unique biology that can have three etiologies: sporadic, familial, and, in some instances, infectious [1]. In the classical prion diseases (referred to here as Prion), which include Creutzfeldt-Jakob disease (CJD) in humans and scrapie in sheep and goats, disease is caused by misfolding of the prion protein (PrP) into PrP^Sc^, a self-templating conformation of the normal host protein PrP^C^. It is now understood that PrP is not the only protein that uses this disease mechanism; additional proteins including β-amyloid, tau, and α-synuclein also misfold into β-sheet-rich conformations that self-template (referred to here as prion), leading to neurodegenerative disorders including Alzheimer’s disease (AD), progressive supranuclear palsy (PSP), and multiple system atrophy (MSA) [2,3]. Within prion diseases, the ability of a single protein to cause multiple distinct clinical disorders is explained by the strain hypothesis, or the concept that the conformation the protein misfolds into dictates the resulting disease [4]. Prion strains are operationally defined as a heritable phenotype of disease under defined experimental conditions, including incubation period, clinical signs of disease, cellular and tissue tropism, and host range [5]. Moreover, each strain is distinguished by biochemical differences that include migration on SDS-PAGE, resistance to proteolytic digestion, solubility in detergents, and conformational stability. Recent cryo-electron microscopy (cryo-EM) data has bolstered the strain hypothesis showing that the protein fibrils isolated from distinct patient cohorts and prion strains contain strain-specific parallel in-register intermolecular β-sheet structures [2,6].

## Prion strains exist as mixtures

**Fig 1:**
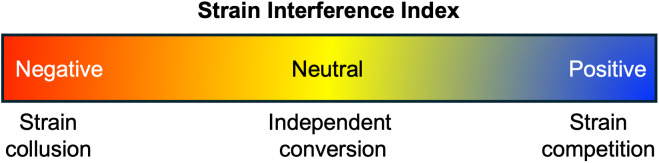
The strain interference index. Prion strains, when present in the same host, can either compete with each other to inhibit propagation, replicate independently, or collude with one another to accelerate disease onset faster than either strain alone. The Strain Interference Index is a hypothetical continuum, or scale, of possible strain interference combinations that range from strong interference (positive index value) to low interference with high synergistic interactions (negative index value).

Strong evidence exists for the co-existence of more than one PrP^Sc^ strain in an individual. For example, sheep scrapie is categorized into classical and atypical strains, which are identified by electrophoretic migration of the unglycosylated PrP^Sc^ polypeptide to 21 kDa (Type 1) for classical scrapie and 19 kDa (Type 2) for atypical scrapie [7]. An individual affected sheep can contain PrP^Sc^ with biochemical characteristics of both the classical and atypical strains, indicative of coinfection. Similarly, in humans, distinct sporadic CJD (sCJD) strains are also distinguished by differences in the migration of PrP^Sc^. Moreover, a mixture of both type 1 and type 2 PrP^Sc^ are present in up to 40% of sCJD cases tested. However, strains with similar biochemical features and/or strains at low abundance are not readily detected within mixtures [8]. For example, α-synuclein fibrils from MSA patient samples have a consistent biochemical stability to denaturants [9], but multiple fibril conformations have been resolved by cryo-EM [10]. This includes the recent isolation of a low-abundance conformation (5% of filaments) present in a patient sample containing multiple fibril structures [11]. A noteworthy consequence of strain mixtures is the rapid emergence of drug resistance, which led to the hypothesis that PrP^Sc^ strains exist as quasispecies. In a prion quasispecies, strains exist as mixtures of a dominant conformation along with minor subpopulations or substrains. These minor populations are likely important in prion ecology, allowing for rapid adaptation to a new replication environment (e.g., an anti-Prion drug or a new host species). Overall, converging lines of evidence indicate that prions exist as a mixture of a dominant and minor strains. The following sections will focus on the biological consequences of strain mixtures, introducing the concept of the Strain Interference Index (Fig 1).

## Consequences of prion strain mixtures: PrP^Sc^ strain competition

The first biological consequence of strain mixtures identified was competition, which is represented as a positive index on our Interference Scale (Fig 1, Table 1). This effect was first observed in mice where inoculation of a long incubation period (i.e., blocking) PrP^Sc^ strain prior to superinfection with a short incubation period PrP^Sc^ strain either extended the incubation period or completely blocked the superinfecting strain from causing disease, thus exerting a *strong interfering effect* on PrP^Sc^ formation [12]. It is now known that competition occurs by a variety of PrP^Sc^ strain combinations, following superinfection or co-infection (e.g., staggered or simultaneous infection), and by neuronal and extraneuronal routes of infection in both mice and hamsters [13]. In this paradigm, strain dominance is dependent on the relative rates of PrP^Sc^ formation between the two strains in a common population of cells. The field has started to elucidate the mechanisms underlying strain competition, showing that the ability of the blocking strain to interfere with the superinfecting strain is not due to a host response to the blocking strain (e.g., interferon production or destruction of cells required for infection by the superinfecting strain). This is supported by the observation that PrP^Sc^ strain competition is recapitulated using protein misfolding cyclic amplification (PMCA). As PMCA is a cell-free system that supports PrP^Sc^ formation, these data suggest that competition occurs at the level of PrP^C^ to PrP^Sc^ conversion. Subsequent studies found that combining strains with long incubation periods or with relatively slow PrP^Sc^ formation kinetics results in independent amplification (e.g., *neutral strain interaction*) in both animals and via PMCA, suggesting interference occurs when PrP^Sc^ strains compete for PrP^C^ [14]. Overall, orthogonal lines of investigation indicate that prions are a dynamic mixture of strains that can compete under the appropriate conditions.

**Table 1:** Interference index examples and outcomes.

Interference Index	Interaction	Outcome	Example
Positive	One strain outcompetes another for access to monomeric protein	Faster replicating strain inhibits propagation of the slower strain	Dickinson and colleagues (1972) [12]
Neutral	Two strains simultaneously replicate in the same cell	Both strains propagate without altering kinetics of the other	Eckland and colleagues (2018) [14]
Negative	The presence of a second strain accelerates kinetics of a single strain	Both strains propagate faster than either would individually	Holec and colleagues (2025) [15]

## Consequences of prion strain mixtures: Synergy

Recent studies aimed to investigate the ability of two α-synuclein strains to compete with one another using the superinfection paradigm discussed above [15]. Unexpectedly, rather than finding that the blocking strain inhibited the shorter incubation strain, superinfection resulted in a synergistic acceleration of disease onset. Not only did mice develop clinical disease faster than injection with the faster strain only, but cell-based assays detected evidence of both the faster and slower replicating strains in the brains of symptomatic animals. These findings indicate that the consequence of strain interference in this case was the exacerbation of not just one α-synuclein strain, but that disease kinetics of both strains were accelerated (Table 1), leading to the term “strain collusion” to describe the novel interaction. As a result, this is shown as a *negative index* on our Strain Interference Scale (Fig 1). The mechanism(s) underlying strain collusion are completely unknown and may include alterations of the pathogenesis of disease, prion formation, or neurodegeneration. One plausible explanation is that co-infection results in proteostasis collapse, allowing both strains to replicate without interference from cellular homeostasis machinery [16]. Alternatively, these experiments used transgenic mice that overexpress human α-synuclein at 3-fold levels greater in the brain and almost 20-fold greater in the spinal cord [17] which could reduce the replication bottleneck created by limited access to monomeric α-synuclein, as likely occurs in strain competition, allowing both strains to propagate efficiently. Finally, this latter explanation could also be compounded by proteostasis collapse. Regardless of mechanism, ongoing studies are focused on determining if the outcome of strain collusion is truly a strain mixture, if a novel strain emerges in terminal animals, or if this can occur with other proteins. Overall, these experiments indicate that interference between prion strain mixtures can range from a positive interaction (competition) to a negative one (collusion).

## Therapeutic implications of prion strain dynamics

Therapeutic interventions are known to affect strain dynamics. For example, efforts to identify small molecules that interfere with PrP^Sc^ formation resulted in the emergence of drug-resistant PrP^Sc^ strains. However, removal of treatment led to rapid reversion back to the drug sensitive phenotype. Consistent with the quasispecies hypothesis, these data suggest that targeted inhibition of the major PrP^Sc^ strain enables emergence of preexisting minor strains or substrains [18]. Other therapeutic interventions have focused on reducing the amount of host-encoded template available for conversion (e.g., anti-sense oligonucleotides), given that genetic ablation of the monomeric protein is protective against disease. Consistent with this observation, a reduction in substrate levels results in a delayed disease onset, alteration of the pathogenesis of disease, and, in some cases, reversal of pathology [19,20]. However, given that prion strains can exist as quasispecies, reducing the amount of protein available for conversion may alter strain interactions, and, consequently, shift strain dynamics. For example, changing the abundance of PrP^C^ may alter strain interaction conditions (i.e., shift from a negative to positive index, or vice versa), which could result in the emergence of a different minor strain or substrain. As efforts continue to focus on developing disease-altering therapeutics for protein misfolding diseases, it is critical that the field simultaneously investigate the effect of each strategy on strain dynamics and interactions.

## Future directions

Interference between prion strains range from competition (positive index) to collusion (negative index), with independent conversion (neutral index) positioned between the two extremes (Fig 1). What remains unknown are the factors that determine how strains interact with one another and how strain mixtures, or quasispecies, influence strain dynamics. This latter question is particularly important when considering ongoing efforts to develop therapeutics. Therapeutic strategies in use or development largely rely either on small molecules or antibodies to inhibit prion formation, or vaccines and other modalities to reduce the amount of template available for conversion. Understanding the parameters that influence strain interference—from competition to collusion—will enable therapeutic strategies that avoid unintended consequences, such as drug resistance, through altered strain dynamics.
